# Tooth loss in Brazilian adolescents: a multilevel cross-sectional study

**DOI:** 10.1590/1980-549720260007.supl.1

**Published:** 2026-05-29

**Authors:** Lara de Souza Cruz Lizardo, Rafaela da Silveira Pinto, Widla Emanuella Pereira Barreto Garcez, Jacqueline Silva Santos, Renata Castro Martins, Mauro Henrique Nogueira Guimarães de Abreu

**Affiliations:** I Universidade Federal de Minas Gerais, School of Dentistry, Department of Social and Preventive Dentistry – Belo Horizonte (MG), Brazil.; II Secretaria de Estado de Saúde de Minas Gerais, Subsecretaria de Redes de Atenção à Saúde, Superintendência de Atenção Primária à Saúde, Diretoria de Políticas de Atenção Primária em Saúde, Coordenação de Saúde Bucal e Ações Integradas – Belo Horizonte (MG), Brazil.

**Keywords:** Adolescent, Tooth loss, Oral health, Adolescent health, Dental health surveys, Dental health services

## Abstract

**Objective:**

To investigate factors associated with tooth loss among Brazilian adolescents aged 15 and 19 years.

**Methods:**

This is a cross-sectional study conducted based on secondary data from the National Survey of Oral Health (SB Brasil 2023), carried out by the Ministry of Health. Variables were analyzed at the individual (sociodemographic characteristics, oral morbidity, and use of dental services), municipal, and state levels. Multilevel Poisson regression models with robust variance were developed to estimate unadjusted and adjusted prevalence ratios. The present study included 8,053 adolescents who participated in the SB Brasil 2023.

**Results:**

The prevalence of tooth loss in this population was 13.77%. Individual and contextual factors were associated with this condition. Each one-year increase in age was associated with a 19% higher prevalence of tooth loss. Higher prevalence was identified among beneficiaries of social programs (25%), adolescents reporting toothache (66%), and users of public health services (23%) compared with those using private services. Greater oral health coverage in Primary Health Care, Gross Domestic Product, and Human Development Index were associated with lower prevalence of the outcome.

**Conclusion:**

Tooth loss among Brazilian adolescents aged 15 to 19 years remains a condition influenced by sociodemographic factors, socioeconomic inequalities, oral morbidity, and predominantly curative access to dental services. Our findings highlight the need to strengthen preventive and equitable actions, especially for vulnerable populations, to reduce regional disparities and promote better oral health outcomes at the national level.

## INTRODUCTION

The high occurrence of dental caries and periodontal diseases in the Brazilian population indicates that oral illness follows a cumulative and socially determined pattern^1,2^. In this context, tooth loss is an outcome of epidemiological interest, as it reflects individual and contextual exposures throughout life^3^, demonstrating special relevance for analytical approaches that incorporate multiple levels of determination^4^.

Considering this epidemiological reality, the World Health Organization (WHO)^5^ establishes that 20 permanent teeth consist in the minimum number necessary to ensure that individuals do not have an impaired social life and masticatory dysfunction^4^. Although the evolution of dentistry has significantly contributed to the reduction of oral health problems in the last decades^6,7^, the persistence of dental loss as an outcome, even in contexts of increasing access to dental services^1,2^, evidences structural limits of public oral health policies in overcoming historically-created inequalities^8-10^. When observed in adolescents, tooth loss bears significant epidemiological relevance^4^ because it occurs in a stage of life in which permanent dentition is expected to be maintained, being an indicator of accumulated failures in prevention, timely care, and longitudinal follow-up processes^11-13^.

The National Survey of Oral Health (*Pesquisa Nacional de Saúde Bucal* – SB Brasil), carried out in 1986, 2003, 2010, and 2023, constitutes a population-based epidemiological survey that monitor the oral health conditions of the Brazilian population, providing fundamental subsidies for the planning of public policies. In its latest edition, SB Brasil 2023 provides up-to-date and representative data on caries experience and its clinical consequences in adolescents aged 15 to 19 years, including tooth loss measured by the DMFT (Decayed, Missing, and Filled Teeth) index^1^. This survey broadens the investigative potential by enabling the individualized analysis of the "missing" component in this age group, in a recent epidemiological scenario, characterized by changes in public oral health policies and in the organization of services.

Adolescence is marked by behavioral and psychosocial changes and greater autonomy in food choices, linked to social and family conditions^12-14^. Tooth loss in this age group is a particularly relevant indicator, as it reflects not only the experience of accumulated caries, but also failures in prevention systems and access to dental services^8,13^, pointing to limitations in the effectiveness of actions aimed at health prevention and promotion as well as the persistence of curative-centered care practices^6^.

Tooth loss in adolescents may have a multifactorial etiology, being potentially influenced by different determinants that operate at individual and collective levels. At the individual level, aspects — such as health behaviors, family socioeconomic conditions, and access to dental care — can determine the risk of tooth loss. Likewise, characteristics of the context where adolescents live, including the availability of dental services, municipal socioeconomic conditions, and public oral health policies, may have a complementary influence on this outcome^2^. Being aware of these factors, in a recent population-based study, has a prominent significance for the Brazilian Unified Health System (SUS), enabling advances in actions for health promotion and care. Despite the recognition that individual and contextual factors influence tooth loss in adolescents, studies whose authors simultaneously deepen these determinants are still limited, especially based on recent national surveys and multilevel approaches. Hence, in this study, we aimed to carry out a multilevel analysis investigating factors associated with tooth loss in Brazilian adolescents aged 15 to 19 years.

## METHODS

### Study design

This is a cross-sectional study based on secondary data from the National Survey of Oral Health 2023, conducted by the Brazilian Ministry of Health.

### Sample

For the sample design, the 27 Federative Units (FU) (26 states and the Federal District) were considered as study domains, in addition to the capitals, totaling 53 geographic domains (capitals and cities on the outskirts of each state, distributed among the five regions of the country: North, Northeast, Midwest, Southeast, and South). The population was organized into 53 strata: Federal District, capitals, and municipalities on the outskirts of each FU. In each stratum, census tracts were drawn and, subsequently, households. For the index ages of 5 and 12 years, sampling was carried out in a single stage — all households drew in these sectors were visited. Complete details about the sampling process can be verified in the SB Brasil 2023 report^1^, as well as, in a summarized way, in the study conducted by Alves et al.^15^. For the present study, all adolescents aged 15 to 19 years, whose ages were registered in years, were included. Of a sample of 8,054 adolescents in this age group, 8,053 individuals were included, distributed in 345 municipalities in the 27 Brazilian states. One individual without age registration in years was excluded.

### Variables

Data were obtained through interviews with questionnaires containing information on demographic, socioeconomic, and individual characteristics, including access to and use of dental services, morbidity, self-rated oral health, and impact on daily activities, as well as clinical examinations evaluating dental caries. Additional information on the collection and calibration of the examiners is available in the official survey report and in Ferreira et al.^16^.

Tooth loss was defined as the absence of at least one permanent tooth due to caries (missing component — "M" of the Decayed, Missing, and Filled Teeth index –DMFT – equal to or greater than one, M≥1). As independent variables, the individual variables collected in SB Brasil 2023 (Level I) were considered, namely: sociodemographic (age, sex, race/skin color, level of education, forms of access to water, Internet access, beneficiary of social programs, and coverage by dental health insurance), oral morbidity (self-reported toothache), and use of services (type of oral health service used). All variables of the individual level were dichotomized, except age (quantitative) and type of service used (polytomous). Independent variables were evaluated at municipal (Level II) and state (Level III) levels. At the municipal level, variables on the organization of the work process in Primary Health Care (PHC) were measured, involving indicators of access, problem-solving capacity, and preventive procedures performed. Indicators of organization of the oral health care network and sociodemographic indicators were also included. At the state level, in turn, the Human Development Index (HDI) and the Gini Index were measured^2,6,8,13^. Covariates of the municipal and state levels were quantitative, except population size (ordinal categorical) and existence of Dental Specialty Centers (dichotomous). The descriptions and data sources of the covariates are detailed in Supplementary Material 1.

### Statistical analysis

Initially, descriptive analyses of the outcome and covariates were performed at individual, municipal, and state levels. Crude Poisson regression models with robust variance were developed to test the association between independent variables at the individual level and tooth loss as a binary variable. Crude prevalence ratios (PR) (95% confidence intervals – 95%CI) and p-values were estimated. To verify the need for development of multilevel regression models, the null model was developed. Multilevel Poisson regression analysis was used to estimate PR and 95%CI among the individual, municipal, and state variables in relation to the binary outcome. Poisson regression models with robust variance can be used to estimate PR in cross-sectional studies with the binary dependent variable^17^. The first model included individual variables with p<0.20 in the crude regression models; the second, municipal variables and individual covariates with p<0.20. In turn, the final model included the state covariates, maintaining those with p<0.20 in the previous models.

Explained variance at the municipal and state levels was estimated by the variance partition coefficient, comparing the final model to the null model. Primary analyses were carried out with individuals who presented all the aforementioned data. Sensitivity analyses were also performed, including the category "do not known/did not answer" for each covariate of the individual level. Statistical analyses, considering sample weights, were carried out using SPSS 23.0 and *STATA* 12.0 (College Station, TX, USA).

### Ethical aspects

The SB Brasil 2023 Project followed Resolution No. 466/2012, of the National Health Council (CNS), and was approved by the National Commission of Ethics in Research (Conep), under CAAE 34497120.6.3001.0008 and Opinion No. 4.823.054, and participants signed the Informed Consent Form.

#### Data Availability Statement:

Data are available upon request.

## RESULTS

The description of the demographic conditions and oral morbidity of the 8,053 adolescents participating in the SB Brasil 2023 national survey is described in Table 1. The contextual variables at the municipal and state levels are described in Supplementary Materials 2 and 3, respectively. The prevalence of tooth loss was 13.77% (95%CI 11.93–15.85). In the crude analysis, tooth loss was more frequent among non-whites, beneficiaries of social assistance programs, adolescents with toothache in the last six months, and users of the public dental service (p<0.05) (Table 2).

**Table 1:** Frequency of individual independent variables surveyed among Brazilian adolescents aged 15 to 19 years (n=8,053), 2023.

Dimension		Variable	n	% (95%CI)
Sociodemographic	Sex			
		Women	4,178	49.81 (46.89–52.72)
		Men	3,875	50.18 (47.27–53.10)
	Race/skin color			
		White	2,604	37.73 (33.11–42.58)
		Non-white	5,359	61.51 (56.69–66.12)
		Do not know/did not answer*	90	0.75 (0.42–1.31)
	Forms of access to water			
		Piped water	7,438	93.97 (92.06–95.45)
		Not piped	226	2.67 (1.73–4.09)
		Do not know/did not answer*	389	3.34 (2.29–4.84)
	Internet access			
		No	349	3.27 (2.49–4.28)
		Yes	7,385	93.43 (91.58–94.90)
		Do not know/did not answer*	319	3.29 (2.23–4.81)
	Beneficiary of social assistance programs			
		No	4,030	50.44 (46.25–54.62)
		Yes	3,293	41.51 (37.65–45.48)
		Do not know/did not answer*	609	8.03 (5.33–11.94)
	Coverage by dental health insurance			
		No	6,997	86.25 (82.08–89.57)
		Yes	910	12.03 (8.83–16.19)
		Do not know/did not answer*	146	1.70 (0.61–4.64)
			**Average (95%CI)**	
	Age (years)		16.85 (16.77–16.93)	
	Level of education (years)		11.03 (10.62–11.45)	
Oral morbidity	Toothache in the last six months			
		No	6,289	77.70 (74.79–80.36)
		Yes	1,708	20.93 (18.59–23.49)
		Do not know/did not answer*	56	1.35 (0.57–3.16)
Use of services	Type of service used			
		Others (private, insurance plans, others)	3,358	45.10 (41.18–49.08)
		Public	3,784	43.91 (40.59–47.28)
		Have never used them	480	4.59 (3.63–5.79)
		Do not know/did not answer*	431	6.38 (4.01–10.01)

95%CI: 95% Confidence interval.

* The category "Do not know/did not answer" was considered missing datum in subsequent models.

**Table 2:** Individual factors associated with tooth loss among Brazilian adolescents aged 15 to 19 years, 2023.

Dimension		Variable	Tooth loss (%, 95%CI)	Crude PR	p-value
Sociodemographic	Sex				
		Women	13.88 (11.48–16.70)	1	0.642
		Men	13.67 (10.84–17.11)	0.97 (0.87–1.09)	
	Race/skin color				
		White	9.42 (7.27–12.12)	1	<0.001
		Non-white	16.57 (14.16–19.29)	1.35 (1.17–1.56)	
	Forms of access to water				
		Piped water	13.54 (11.66–15.66)	1	0.013
		Not piped	16.86 (9.47–28.21)	1.47 (1.08–1.98)	
	Internet access				
		No	17.50 (11.81–25.14)	1	0.005
		Yes	13.54 (11.64–15.70)	0.70 (0.55–0.90)	
	Social assistance programs				
		No	10.33 (8.22–12.91)	1	<0.001
		Yes	17.79 (14.99–20.97)	1.43 (1.26–1.63)	
	Health insurance coverage				
		No	14.60 (12.59–16.87)	1	0.009
		Yes	8.77 (5.87–12.92)	0.75 (0.61–0.93)	
	Age			1.18 (1.13–1.23)	<0.001
	Level of education			1.00 (0.99–1.01)	0.822
Oral morbidity	Toothache in the last six months				
		No	10.67 (9.11–12.47)	1	<0.001
		Yes	24.75 (19.01–31.54)	1.89 (1.67–2.15)	
Use of services	Others (private, insurance plans)		13.92 (7.54–24.29)	1	
		Public	17.50 (14.47–21.02)	1.37 (1.21–1.56)	<0.001
		Have never used them	10.07 (7.96–12.65)	0.65 (0.47–0.91	0.013

95%CI: 95% Confidence interval; PR: Prevalence ratio.

The multilevel model with individual, municipal and state variables was developed with 6,854 adolescents (missing rate due to data incompleteness=14.9%). According to our findings, individuals beneficiaries of social programs had a 25% higher prevalence of tooth loss compared to non-beneficiaries (PR 1.25; 95%CI 1.10–1.43). Each one-year increase in age increases the prevalence of tooth loss by 19% (PR 1.19; 95%CI 1.13–1.24). Presence of toothache increases the prevalence of tooth loss in this group by 66% (PR 1.66; 95%CI 1.45–1.90). Users of public services have a 23% higher prevalence of tooth loss than those using private services (PR 1.23; 95%CI 1.07–1.41). Adolescents who have never used oral health services have a 43% lower prevalence of tooth loss (PR 0.57; 95%CI 0.39–0.83).

At the municipal level, the 1% increase in oral health coverage in PHC decreases the prevalence of tooth loss by approximately 0.5% (PR 0.99; 95%CI 0.99–0.99). The increase in the municipal GDP decreases the prevalence of tooth loss (PR 0.99; 95%CI 0.99–1.00). The prevalence of tooth loss increases as population size decreases, suggesting the existence of a dose-response gradient. In states with higher HDI, the prevalence of tooth loss decreases among Brazilian adolescents (PR 0.04; 95%CI 0.00–0.98).

Assessing changes in variances in the different models compared to the null model allows us to state that the gradual inclusion of variables in the models 1, 2, and 3 improves the percentage of explanation of tooth loss by the selected factors. We observed that the inclusion of the variables of Levels II and III concomitantly explains approximately 100 and 72.04% of the variance, respectively (Table 3).

**Table 3:** Individual, municipal, and state factors associated with tooth loss among adolescents aged 15 to 19 years, Brazil, 2023.

Level I – Individuals							
Variable		Model 1		Model 2		Model 3	
		PR (95%CI)	p-value	PR (95%CI)	p-value	PR (95%CI)	p-value
Race/skin color							
	White	1	0.025	1	0.019	1	0.055
	Non-white	1.21 (1.02–1.42)		1.21 (1.03–1.43)		1.17 (1.00–1.37)	
Forms of access to water							
	Piped water	1	0.189	1	0.261		
	Not piped	1.26 (0.89–1.77)		1.21 (0.87–1.70)			
Internet access							
	No	1	0.082	1	0.132	1	0.059
	Yes	0.79 (0.60–1.03)		0.81 (0.62–1.06)		0.77(0.59–1.01)	
Beneficiary of social assistance programs							
	No	1	0.001	1	0.002	1	0.001
	Yes	1.27 (1.11–1.45)		1.24 (1.09–1.42)		1.25 (1.10–1.43)	
Coverage by dental health insurance							
	No	1	0.161	1	0.228		–
	Yes	0.84 (0.66–1.07)		0.86 (0.68–1.10)			
	Age	1.19 (1.13–1.24)	<0.001	1.18 (1.13–1.24)	<0.001	1.19 (1.14–1.24)	<0.001
Toothache in the last six months							
	No	1	<0.001	1	<0.001	1	<0.001
	Yes	1.67 (1.45–1.91)		1.66 (1.45–1.90)		1.66 (1.45–1.90)	
Type of service used							
	Other types of service (private, insurance plans, others)	1		1		1	
	Public	1.22 (1.06–1.41)	0.006	1.22 (1.06–1.41)	0.007	1.23 (1.07–1.41)	0.003
	Have never used them	0.59 (0.40–0.87)	0.007	0.58 (0.39–0.84)	0.005	0.57 (0.39–0.83)	0.004
**Level II – Municipalities**							
First consultation		–	–	0.98 (0.96–1.00)	0.072	0.98 (0.96–1.00)	0.062
CT/FC		–	–	0.99 (0.99–1.00)	0.310		
Preventive procedures		–	–	0.99 (0.99–1.00)	0.133	0.99 (0.99–1.00)	0.141
OH coverage in PHC		–	–	0.99 (0.99–1.00)	0.064	0.99 (0.99–0.99)	0.038
Existence of CEO							
	No	–	–	1			
	Yes	–	–	0.95 (0.87–1.04)	0.263		
	GDP per capita	–	–	0.99 (0.99–0.99)	0.002	0.99 (0.99–1.00)	0.036
Population size (thousand)							
	>500	–	–	1		1	
	>100 and ≤500	–	–	1.22 (0.95–1.57)	0.114	1.32 (1.08–1.63)	0.007
	>10 and ≤ 100			2.06 (1.33–3.20)	0.001	2.33 (1.70–3.20)	<0.001
	Up to 10	–	–	2.32 (1.25–4.31)	0.008	2.61 (1.62–4.21)	<0.001
**Level III – States**							
HDI		–	–	–	–	0.04(0.00–0.98)	0.048
Gini		–	–	–	–	5.43(0.44–68.74)	0.188
**Random effects**		**Null Model Level II**	**Null Model Level III**	**Null Model Levels II and III**	**Model 1**	**Model 2**	**Model 3**
Variance (95%CI)		0.217 (0.130–0.362)	0.127 (0.065–0.250)	II: 0.099 (0.044–0.223) III 0.142 (0.066–0.309)	II: 0.058 (0.017–0.204) III: 0.098 (0.041–0.236)	II: 0.000 (0.000–0.000) III: 0.063 (0.025–0.158)	II: 0.000 (0.000–0.000) III: 0.039 (0.014–0.110)
LR test (χ^2^; p-value)		74.53 (<0.001)	87.51 (<0.001)	100.07 (<0.001)	46.71 (<0.001)	21.86 (<0.001)	12.35 (0,002)
Variation changes in relation to the null model with levels II and III					II: 41.47% III: 31.19%	II: 99.78% III: 57.62%	II: 100% III: 72.04%

PR: Prevalence ratio; CI: Confidence interval; CT/FC: Proportion of completed treatments in relation to the first consultations; OH: Oral health; PHC: Primary Health Care; CEO: Dental Specialty Centers; HDI: Human Development Index; LR: Likelihood ratio.

As per the sensitivity analysis carried out with the inclusion of the category "do not know/did not answer," we identified the permanence of associations with the variables age, being a beneficiary of social program, toothache, type of service used, and size of the municipality — maintaining the direction of these associations and magnitude of associations close to the model presented in the primary results. There was also the inclusion of new associations (Internet access and indicator of first programmatic dental consultation) and the loss of association (coverage of SB in PHC, GDP, and HDI — which had a marginal association in the original model with p=0.048). In addition, there was a change in model 3 (final) as for the percentage of the explained variance to approximately 95.75 and 76.24%, respectively (Supplementary Material 4).

## DISCUSSION

In this multilevel population-based study, we identified individual and contextual determinants of tooth loss in Brazilian adolescents aged 15 to 19 years. The prevalence of tooth loss remains frequent in this age group, indicating an early and potentially preventable condition, mainly determined by factors of sociodemographic, socioeconomic, oral morbidity, use of services, individual, and contextual nature.

At the individual level, toothache stood out as a relevant factor, increasing the prevalence of tooth loss by 66% and acting as a marker of the severity of caries and motivating the demand for dental care^18^. In the Brazilian context, the perception of toothache and tooth loss influences oral health behavior^19^, being associated with worse outcomes and access barriers, especially in emergency-centered care models, to the detriment of preventive actions^11,19^.

The one-year increase in age increased the prevalence of tooth loss by 19%, corroborating Motta et al.^4^ and possibly reflecting greater severity of caries in older age groups of adolescence as well as cultural factors and access to services. Although Drummond et al.^7^ point to progressive reduction of tooth loss among adolescents in the SB Brasil surveys of 2003, 2010, and 2023, probably due to public policies — such as *Programa Brasil Sorridente* [Smiling Brazil Program] and the Family Health Strategy (FHS) —, considering our findings, we suggest that this outcome still persists, with early accumulation of oral diseases and persistence of regional and socioeconomic inequalities.

The literature on oral health among beneficiaries of cash transfer programs, such as *Bolsa Família* ^20^, is still scarce. Beneficiary adolescents presented 25% higher prevalence of tooth loss, reinforcing the influence of socioeconomic inequalities. Colvara et al.^21^ observed a trend of a higher number of extractions in municipalities with higher program coverage. These findings highlight the need for studies whose authors evaluate this association between different age groups and the persistence of structural barriers and inequalities in oral health access.

Despite the expansion of public policies, such as the National Oral Health Policy (*Política Nacional de Saúde Bucal* – PNSB), known as "Smiling Brazil"; and the FHS, with the incorporation of Oral Health Teams (OHTs), social programs and the expansion of dental services, access and use of dental services remain unequal^22^. This inequality is evidenced by the association between use of services and tooth loss among adolescents, mainly mediated by individual socioeconomic conditions^13,23^.

In the present study, users of the public sector presented 23% higher prevalence of tooth loss, while those who never used dental services presented 43% lower prevalence. These results should be interpreted with caution, as the lower prevalence among adolescents who have never used dental services may reflect a need-oriented pattern of use, compatible with the bias of the healthy individual, in which individuals with better oral health conditions tend to demand less health services^8,24^.

According to these findings, we can infer that the use of public service occurs, overall, in the face of accumulated needs, with a predominant search for pain, urgency, and/or curative demands, and not for preventive actions, reflecting a model historically centered on urgency and emergency care^21^. Authors of previous studies corroborate this finding, such as Motta et al.^4^, who found a higher frequency of consultations motivated by pain on a study on adolescents seen at the SUS; while Oliveira et al.^10^ highlighted that using SUS services is associated with more unfavorable socioeconomic conditions and less access to preventive care.

Adolescents living in municipalities with higher oral health coverage in PHC presented lower prevalence of tooth loss, reinforcing the effectiveness of actions on health prevention and promotion at this level of care. This finding reinforces the strategic performance of PHC as coordinator of care and gateway to the SUS. Structured mainly through the FHS and OHTs, PHC has an essential role in preventing caries and reducing its consequences — such as tooth loss^25-27^. Thus, the greater coverage of PHC reflects greater capacity of municipalities to organize preventive responses and actions, reduce inequalities, and promote comprehensive care.

It is worth emphasizing that the results found at the individual level (users of the public sector) do not contradict the findings of contextual indicators (such as PHC coverage), as each reflects different mechanisms. The use of public service tends to reflect established needs, while the coverage of PHC and other municipal factors demonstrate the organizational capacity of the territory to offer preventive actions and coordinate care. The category "Have never used dental services" should be interpreted with caution, as it may reflect both absence of perceived need and barriers to access services, influencing the observed associations.

The increase in municipal GDP decreased the prevalence of tooth loss, suggesting that local economic development can provide better living conditions and access to health resources. However, this result should be interpreted with caution. As an aggregate macroeconomic indicator, GDP does not capture the distribution of income in the territory, and can coexist as a concentration of wealth and inequalities in access to services^28,29^. Thus, the finding may reflect structural characteristics of the territory, but it does not mean that there is better distribution or quality of care. For a more comprehensive interpretation, other inequality indicators should be considered.

Regarding the population size of the municipalities, those of larger size tend to present greater availability of health services and professionals, factors that facilitate access to oral health care and contribute to better outcomes^12^. Conversely, small municipalities often deal with challenges related to the availability of resources and the hiring of professionals, which tends to compromise the supply and quality of dental care^30^.

HDI has a strong correlation with health indicators, demonstrating that the health-disease process also results from quality-of-life conditions^31,32^. According to our results, states with higher HDI were associated with lower prevalence of tooth loss among Brazilian adolescents, suggesting that better socioeconomic and educational conditions reflect on more favorable oral health outcomes^9^. Authors of previous studies conducted with Brazilian adults identified lower averages of missing teeth and lower socioeconomic inequalities in municipalities with higher HDI^9,33^. Nonetheless, because it is an aggregate indicator, the state HDI can mask intraregional and local inequalities, indicating the need for municipal analysis to improve the understanding of the contextual and territorial effects associated with tooth loss^34^.

Among the variables analyzed in this study, we observed no statistically significant associations (p<0.05) between tooth loss and the following individual characteristics: forms of access to water, Internet access, and coverage by dental health insurance. At the municipal level, coverage of first dental consultation, proportion of completed treatments, preventive procedures, and existence of Dental Specialty Centers (*Centros de Especialidades Odontológicas* – CEO) did not show any association with the outcome. Finally, at the state level, we found no significant association between the Gini index (which expresses income inequality in the population) and tooth loss among adolescents. This finding may be a positive consequence of public policies to increase PHC and the fairer supply of dental services in the country, which contribute to reducing regional inequalities in oral health. Authors, such as Ferreira et al.^9^ and Roberto et al.^33^, point out that the cumulative effect of social determinants tends to intensify in more advanced age groups, which would explain the smaller influence of these factors on the population of adolescents aged between 15 and 19 years.

Tooth loss is strongly associated with poor quality of life, affecting daily activities and social, emotional, and psychological well-being^3,35^. In adolescence, the impact is maximized because it is a phase of greater vulnerability, marked by biopsychosocial transformations and risk behaviors, such as smoking, alcohol consumption, and inadequate oral hygiene practices, which can perpetuate in future life^14^.

It should be noted that this is a cross-sectional study, making it impossible to establish causality relations between the analyzed variables. In addition, the use of secondary databases may imply limitations related to the presence of missing data and the possible incorrect filling out of information, even those from official databases of the SUS. It is noteworthy that we used data from the most recent National Survey of Oral Health (SB Brasil 2023), which allows the analysis of the most current data about the oral health of the population in Brazil.

As per the sensitivity analysis, findings should be interpreted with caution, in view of the modification of variables associated with the outcome with the inclusion of the category "do not know/did not answer" in the relevant variables. Nevertheless, the variables with more consistent associations remained in the original model and in this sensitivity analysis — which can be considered a positive factor for primary results. To date, no studies have been identified that evaluate tooth loss in Brazilian adolescents aged 15 to 19 years, considering individual and contextual factors based on the most recent national data. The results are relevant because they provide a comprehensive and updated national perspective, contributing to the evaluation of services and the improvement of public policies aimed at expanding access and improving dental care in the country.

Finally, tooth loss among adolescents aged 15 to 19 years remains an important public health issue, strongly influenced by sociodemographic, oral morbidity, access to dental services, and contextual conditions. The results indicate the urgency of managers and governments to strengthen promotion and prevention actions, expand access to dental care, focus on territories with greater inequalities, and prioritize more equitable and integrated public policies. Authors of future studies should compare these findings with other age groups and periods, contributing to the monitoring of temporal trends in oral health in the country.
