# Supplementary material for: Tooth loss in Brazilian adolescents: a multilevel cross-sectional study
Source: Rev Bras Epidemiol. 2026 May 29;29(Suppl 1):e260007supl1. doi: 10.1590/1980-549720260007.supl.1 (PMC13221167; doi:10.1590/1980-549720260007.supl.1)
Supplement: Material Suplementar [file 1980-5497-rbepid-29-suppl1-e260007supl1-supp01.pdf]

Material suplementar 1 – Variáveis independentes e suas fontes de dados no nível individual, municipal e estadual

**Variáveis independentes**				
**Nível I - indivíduos**				
**Dimensão**	**Variável**	**Categoria**	**Fonte de dados**	**Ano**
Sociodemográficas	Idade	Em anos	SB BRASIL	2023
	Sexo	Feminino ; Masculino	SB BRASIL	2023
	Raça/Cor	Branco; Não branco	SB BRASIL	2023
	Escolaridade	Número de anos de estudo	SB BRASIL	2023
	Formas de acesso à água	Canalizada- pelo menos um cômodo ou no terreno; Não Canalizada	SB BRASIL	2023
	Acesso à internet	Não; Sim	SB BRASIL	2023
	Beneficiário de programas de assistência social	Não; Sim	SB BRASIL	2023
	Cobertura por plano de saúde odontológico	Não; Sim	SB BRASIL	2023
Morbidade bucal	Dor de dente nos últimos 6 meses	Não; Sim	SB BRASIL	2023
Utilização de serviços	Tipo de serviço utilizado	Nunca usou; público; outros	SB BRASIL	2023
**Nível II – Municípios**				
Organização do processo de trabalho na Atenção Primária à Saúde	Cobertura de primeira consulta odontológica programática (Primeira Consulta)	Numerador: a) nº total de pessoas com atendimentos de primeira consulta odontológica programática. Denominador: b) nº total de pessoas vinculadas à equipe.	SISAB	2023
	Proporção de tratamentos concluídos em relação às primeiras consultas (TC/PC)	1. Numerador: nº total de pessoas com tratamento odontológico concluído por eSB na APS.<br>2. Denominador: nº total de pessoas com primeira consulta odontológica programada na APS.	SISAB	2023
	Proporção de procedimentos odontológicos preventivos realizados em relação ao total de procedimentos odontológicos individuais (Procedimentos Preventivos).	1. Numerador: nº total de procedimentos odontológicos individuais preventivos registrados por eSB na APS.<br>2. Denominador: nº total de procedimentos odontológicos individuais registrados por eSB na APS.	SISAB	2023
Organização da rede de atenção em Saúde Bucal	Cobertura de Saúde Bucal na APS (Cobertura SB na APS)	Numérico	E-gestor	2023
	Existência de CEO	Não; Sim	CNES	2023

Sociodemográficas	Porte populacional	Até 10 mil habitantes<br>>10 mil e ≤ 100.000 habitantes<br>>100 mil e ≤ 500.000 habitantes<br>>500 mil habitantes	IBGE	2023
	PIB *per capita*	Númerico	IBGE	2021
**Nível III – Estado**				
	IDH	Índice de Desenvolvimento Humano	PNUD	2022
	GINI index	Índice de Gini - estima a desigualdade de renda em uma área de uma determinada população	PNUD	2022

Material Suplementar 2 – Descrição das covariáveis em nível municipal, Brasil.

Variáveis quantitativas			
	**Média (DP)**	**Mediana**	**Mínimo-Máximo**
Percentual de CobPrimConsul* (N=345)	7,89 (8,85)	5,90	0,00-83,82
Percentual de PropTCPC** (N=345)	38,55 (33,38)	40,00	0,00-212,82
Percentual de PropProcPrev*** (N=345)	37,86 (37,84)	33,19	0,00-450,98
PIB per capita em Reais (N=345)	37.699,48 (42.251,15)	26.119,02	7.325,60-511.810,80
Percentual de Cobertura de SB na APS**** (N=345)	74,30 (27,28)	82,52	11,15-100,00
Variáveis categóricas	**Frequência**	**Percentual**	
Presença de CEO			
Não tem CEO	139	40,3	
Só CEO tipo I	49	14,2	
Só CEO tipo II	81	23,5	
Só CEO tipo III	35	10,1	
Mais de um tipo de CEO	41	11,9	
Total	345	100	
Porte populacional			
Até 10 mil habitantes	32	9,3	
De 10.001 até 100 mil habitantes	171	49,6	
De 100.001 a 500 mil habitantes	101	29,3	
500.001 ou mais	41	11,9	
Total	345	100	

*Cobertura de primeira consulta odontológica programática (Primeira Consulta) **Proporção de tratamentos concluídos em relação às primeiras consultas ; ***Proporção de procedimentos odontológicos preventivos realizados em relação ao total de procedimentos individuais; ****Cobertura de Saúde Bucal na APS.

Material Suplementar 3 - Análise descritiva das covariáveis em nível estadual, Brasil

Variáveis			
	**Média (DP)**	**Mediana**	**Mínimo-Máximo**
IDH (N=27)	0,73 (0,04)	0,73	0,68-0,81
GINI (N=27)	0,52 (0,04)	0,53	0,42-0,60

Material Suplementar 4 – Fatores individuais, municipais e estaduais associados à perda dentária entre adolescentes de 15 a 19 anos, Brasil, 2023., com inclusão da categoria "Não sabe/não respondeu".

Nível I – Indivíduos

**Variável**	**Modelo 1** RP (IC95%)	**p-valor**	**Modelo 2** RP (IC95%)	**p-valor**	**Modelo 3** RP (IC95%)	**p-valor**
Raça/Cor						
Branco	1		1		1	
Não Branco	1,16 (1,00-1,35)	0,046	1,17 (1,01-1,35)	0,035	1,14 (0,98-1,31)	0,083
Não sabe/não respondeu	0,78 (0,40-1,53)	0,470	0,76 (0,39-1,48)	0,412	0,75 (0,38-1,46)	0,391
Formas de acesso à água						
Canalizada	1		1		1	
Não canalizada	1,32 (0,98-1,78)	0,071	1,29 (0,96-1,73)	0,094	1,28 (0,95-1,72)	0,102
Não sabe/não respondeu	0,90 (0,57-1,42)	0,660	0,88 (0,56-1,38)	0,577	0,92 (0,58-1,43)	0,673
Acesso à internet						
Não	1		1		1	
Sim	0,74 (0,58-0,95)	0,018	0,76 (0,60-0,97)	0,029	0,76 (0,60-0,97)	0,028
Não sabe/não respondeu	0,78 (0,45-1,35)	0,367	0,80 (0,46-1,38)	0,422	0,78 (0,45-1,35)	0,376
Beneficiário de programas de assistência social						
Não	1		1		1	
Sim	1,33 (1,16-1,51)	0,001	1,31 (1,15-1,49)	<0,001	1,30 (1,14-1,48)	<0,001
Não sabe/não respondeu	1,47 (1,13-1,92)	0,004	1,45 (1,11-1,88)	0,005	1,44 (1,11-1,87)	0,006
Cobertura por plano de saúde odontológico						
Não	1					
Sim	0,87 (0,69-1,09)	0,219	-	-	-	-
Não sabe/não respondeu	0,74 (0,43-1,28)	0,285	-	-	-	-
Idade	1,17 (1,13-1,22)	<0,001	1,17 (1,12-1,22)	<0,001	1,17 (1,13-1,22)	<0,001
Dor de dente nos últimos 6 meses						
Não	1		1		1	
Sim	1,74 (1,54-1,98)	<0,001	1,73 (1,52-1,96)	<0,001	1,74 (1,53-1,98)	<0,001
Não sabe/não respondeu	2,38 (1,37-4,13)	0,002	2,13 (1,25-3,63)	0,005	2,12 (1,24-3,61)	0,006
Tipo de serviço utilizado						
Demais tipos de serviç (privado, planos, outros)	1		1		1	
Público	1,22 (1,07-1,40)	0,004	1,25 (1,09-1,42)	0,001	1,24 (1,09-1,41)	0,001

Nunca usou	0,62 (0,44-0,87)	0,006	0,63 (0,45-0,88)	0,007	0,62 (0,44-0,87)	0,004
Não sabe/não respondeu	1,16 (0,87-1,53)	0,308	1,15 (0,88-1,51)	0,307	1,16 (0,88-1,52)	0,298
**Nível II – Municípios**						
Primeira consulta	-	-	1,00 (0,96-1,00)	0,035	0,98 (0,96-1,00)	0,039
TC/PC	-	-	1,00 (0,99-1,00)	0,287	-	-
Procedimentos preventivos	-	-	1,00 (0,99-1,00)	0,081	1,00 (1,00-1,00)	0,083
Cobertura de SB na APS	-	-	1,00 (0,99-1,00)	0,117	1,00 (0,99-1,00)	0,084
Existência de CEO						
Não	-	-	1		-	-
Sim	-	-	0,99 (0,91-1,08)	0,802	-	-
PIB per capita	-	-	0,99 (0,99-0,99)	0,004	1,00 (1,00-1,00)	0,052
Porte populacional						
>500 mil	-	-	1		1	
>100 mil e ≤ 500.000	-	-	1,29 (1,02-1,64)	0,034	1,28 (1,06-1,55)	0,009
>10 mil e ≤ 100.000			2,32 (1,53-3,52)	<0,001	2,23 (1,65-3,01)	<0,001
Até 10 mil	-	-	2,87 (1,60-5,14)	<0,001	2,69 (1,73-4,19)	<0,001
**Nível III – Estados**						
IDH	-	-	-	-	0,05 (0,00-1,01)	0,051
Gini	-	-	-	-	4,78 (0,43-52,98)	0,203
**Efeitos Randômicos**	**Modelo Nulo Nível II**	**Modelo Nulo Nível III**	**Modelo Nulo Níveis II e III**	**Modelo 1**	**Modelo 2**	**Modelo 3**
Variância (IC95%)	0,217<br>(0,130-0,362)	0,127<br>(0,065-0,250)	II: 0,099<br>(0,044-0,223)<br>III 0,142<br>(0,066-0,309)	II:0,059<br>(0,019-0,183)<br>III:0,085<br>(0,035-0,206)	II:0,005<br>(0,000-2,628)<br>III: 0,046<br>(0,017-0,119)	II: 0,000<br>(0,000-0,000)<br>III: 0,033<br>(0,012-0,092)
LR test (χ^2. p-valor)	74,53 (<0,001)	87,51 (<0,001)	100,07 (<0,001)	46,09(<0,001)	18,31 (<0,001)	12,97 (0,0015)
Mudanças na variação em relação ao modelo nulo com os níveis II e III				II: 40,87%<br>III: 40,20%	II: 94,33%<br>III: 67,93%	II: 95,75%<br>III: 76,24%
