# Supplementary material for: Tooth loss in Brazilian adolescents: a multilevel cross-sectional study
Source: Rev Bras Epidemiol. 2026 May 29;29(Suppl 1):e260007supl1. doi: 10.1590/1980-549720260007.supl.1 (PMC13221167; doi:10.1590/1980-549720260007.supl.1)
Supplement: Translation

# Translation: Perda dentária em adolescentes brasileiros: estudo transversal multinível

**DOI:** 10.1590/1980-549720260007.supl.1.2

**Authors:** Lara de Souza Cruz Lizardo, Rafaela da Silveira Pinto, Widla Emanuella Pereira Barreto Garcez, Jacqueline Silva Santos, Renata Castro Martins, Mauro Henrique Nogueira Guimarães de Abreu

**Affiliations:** I Universidade Federal de Minas Gerais, Faculdade de Odontologia, Departamento de Odontologia Social e Preventiva – Belo Horizonte (MG), Brasil.; II Secretaria de Estado de Saúde de Minas Gerais, Subsecretaria de Redes de Atenção à Saúde, Superintendência de Atenção Primária à Saúde, Diretoria de Políticas de Atenção Primária em Saúde, Coordenação de Saúde Bucal e Ações Integradas – Belo Horizonte (MG), Brasil.

**Keywords:** Adolescentes, Perda de dente, Saúde bucal, Saúde integral do adolescente, Inquérito de saúde bucal, Serviços de saúde bucal

## Abstract

**Objetivo:**

O estudo buscou investigar os fatores associados à perda dentária em adolescentes brasileiros de 15 a 19 anos.

**Métodos:**

Trata-se de um estudo transversal com base em dados secundários provenientes da Pesquisa Nacional de Saúde Bucal (SB Brasil 2023), conduzida pelo Ministério da Saúde. Foram analisadas variáveis nos níveis individual (sociodemográficas, morbidade bucal e utilização de serviços), municipal e estadual. Modelos multiníveis de regressão de Poisson com variância robusta foram desenvolvidos para estimar razões de prevalência não ajustada e ajustada. O presente estudo incluiu 8.053 adolescentes participantes do inquérito nacional SB Brasil 2023.

**Resultados:**

A prevalência de perda dentária nesta população foi de 13,77%. Fatores individuais e contextuais estavam associados a essa condição. O aumento em um ano da idade esteve relacionado a um aumento de 19% na prevalência de perda dentária. Além disso, identificou-se maior prevalência entre beneficiários de programas sociais (25%), aqueles que relataram dor (66%) e entre usuários do setor público de saúde (23%), quando comparados aos atendidos pelo setor privado. Maiores coberturas de saúde bucal na atenção primária, Produto Interno Bruto (PIB) e Índice de Desenvolvimento Humano (IDH) associaram-se a menores prevalências do desfecho.

**Conclusão:**

A perda dentária em adolescentes brasileiros de 15 a 19 anos permanece associada a desigualdades sociodemográficas e socioeconômicas, morbidade bucal e acesso predominantemente curativo aos serviços odontológicos. Os achados evidenciam a necessidade de potencializar ações preventivas e equitativas, especialmente para populações vulneráveis, como estratégia para reduzir as disparidades regionais e melhorar os desfechos de saúde bucal em âmbito nacional.

## INTRODUÇÃO

A elevada ocorrência de cárie dentária e doenças periodontais na população brasileira indica que o adoecimento bucal segue um padrão cumulativo e socialmente determinado^1,2^. Nesse contexto, a perda dentária constitui um desfecho de interesse epidemiológico, por refletir exposições individuais e contextuais ao longo da vida^3^, configurando especial relevância para abordagens analíticas que incorporam múltiplos níveis de determinação^4^.

Considerando essa realidade epidemiológica, a Organização Mundial da Saúde (OMS)^5^ define que 20 dentes permanentes compõem o número mínimo necessário para assegurar que os indivíduos não tenham a vida social e função mastigatória prejudicadas^4^. Embora a evolução da odontologia tenha contribuído significativamente para a redução dos agravos em saúde bucal nas últimas décadas^6,7^, a persistência da perda dentária como desfecho, mesmo em contextos de ampliação do acesso aos serviços odontológicos^1,2^, demonstra limites estruturais das políticas públicas de saúde bucal na superação de desigualdades historicamente construídas^8-10^. Quando observada em adolescentes, a perda dentária assume relevância epidemiológica significativa^4^, por ocorrer em uma fase do curso de vida em que se espera a manutenção da dentição permanente, funcionando como um indicador de falhas acumuladas nos processos de prevenção, cuidado oportuno e acompanhamento longitudinal^11-13^.

As Pesquisas Nacionais de Saúde Bucal (SB Brasil) realizadas em 1986, 2003, 2010 e 2023 constituem levantamentos epidemiológicos de base populacional que monitoram as condições de saúde bucal da população brasileira, fornecendo subsídios fundamentais para o planejamento de políticas públicas. Em sua edição mais recente, o SB Brasil 2023 disponibiliza dados atualizados e representativos sobre a experiência de cárie e suas consequências clínicas em adolescentes de 15 a 19 anos, incluindo a perda dentária mensurada pelo índice CPO-D^1^ (dentes cariados, perdidos e obturados). Esse inquérito amplia o potencial investigativo ao possibilitar a análise individualizada do componente "perdidos" nessa faixa etária, em um cenário epidemiológico recente, caracterizado por transformações nas políticas públicas de saúde bucal e na organização dos serviços.

A adolescência é marcada por mudanças comportamentais e psicossociais e maior autonomia nas escolhas alimentares, articuladas a condições sociais e familiares^12-14^. A perda dentária nesse grupo etário constitui um indicador particularmente relevante, uma vez que reflete não apenas a experiência de cárie acumulada, mas também falhas nos sistemas de prevenção e no acesso aos serviços odontológicos^8,13^, indicando limitações na efetividade das ações preventivas e de promoção da saúde, bem como a persistência de práticas assistenciais centradas no cuidado curativo^6^.

A perda dentária em adolescentes pode apresentar etiologia multifatorial, sendo potencialmente influenciada por diferentes determinantes que operam em níveis individuais e coletivos. No nível individual, aspectos como comportamentos de saúde, condições socioeconômicas familiares e acesso a cuidados odontológicos podem determinar o risco de perda dentária. Simultaneamente, características do contexto onde os adolescentes residem, incluindo disponibilidade de serviços odontológicos, condições socioeconômicas municipais e políticas públicas de saúde bucal, podem exercer influência complementar sobre este desfecho^2^. Conhecer esses fatores, em estudo recente de base populacional, tem um significado de destaque para o Sistema Único de Saúde (SUS), viabilizando avanços nas ações de promoção de saúde e no cuidado em saúde. Apesar do reconhecimento de que fatores individuais e contextuais influenciam a perda dentária em adolescentes, ainda são limitados os estudos que aprofundam simultaneamente esses determinantes, especialmente com base em inquéritos nacionais recentes e com abordagens multiníveis. Sendo assim, este estudo teve como objetivo realizar uma análise multinível investigando os fatores associados à perda dentária em adolescentes brasileiros de 15 a 19 anos.

## MÉTODOS

### Desenho do estudo

Trata-se de um estudo transversal realizado com base em dados secundários da Pesquisa Nacional de Saúde Bucal 2023, realizada pelo Ministério da Saúde.

### Amostra

Para o desenho amostral, foram consideradas as 27 Unidades Federativas (UF) (26 estados e o Distrito Federal) como domínios de estudo, além das capitais, totalizando 53 domínios geográficos (capitais e interiores de cada estado, distribuídos pelas cinco regiões do país: Norte, Nordeste, Centro-Oeste, Sudeste e Sul). A população foi organizada em 53 estratos: Distrito Federal, capitais e municípios do interior de cada UF. Em cada estrato, foram sorteados setores censitários e, em seguida, domicílios. Para as idades-índice de 5 e 12 anos, a amostragem foi em estágio único — todos os domicílios sorteados nesses setores foram visitados. Detalhes completos sobre o processo amostral podem ser consultados no relatório do SB Brasil 2023,^1^ bem como, de forma sintetizada, no estudo de Alves et al.^15^. Para o presente estudo foram incluídos todos os adolescentes de 15 a 19 anos que tiveram o registro da idade em anos. De uma amostra de 8.054 adolescentes nessa faixa etária, foram incluídos 8.053 indivíduos distribuídos em 345 municípios dos 27 estados brasileiros. Um indivíduo sem o registro da idade em anos foi excluído.

### Variáveis

Os dados foram obtidos por entrevistas com questionários sobre informações demográficas, socioeconômicas e características individuais, incluindo acesso e uso de serviços odontológicos, morbidade, autopercepção da saúde bucal e impacto nas atividades diárias, além de exames clínicos avaliando cárie dentária. Informações complementares sobre coleta e calibração dos examinadores estão disponíveis no relatório oficial do inquérito e em Ferreira et al.^16^.

A perda dentária foi definida como a ausência de pelo menos um dente permanente devido à cárie (componente perdido — P do índice de dentes cariados, perdidos e obturados – CPOD – igual ou superior a um, P≥1). Como variáveis independentes, foram consideradas aquelas individuais coletadas no SB Brasil 2023 (Nível I), a saber: sociodemográficas (idade, sexo, raça/cor, escolaridade, forma de acesso à água, acesso à internet, beneficiário de programas sociais e cobertura por plano de saúde odontológico), morbidade bucal (dor de dente autorrelatada) e utilização de serviços (tipo de serviço de saúde bucal utilizado). Todas as variáveis do nível individual foram dicotomizadas, exceto idade (quantitativa) e tipo de serviço utilizado (politômica). Avaliaram-se variáveis independentes nos níveis municipal (Nível II) e estadual (Nível III). No municipal, foram mensuradas variáveis sobre organização do processo de trabalho na Atenção Primária à Saúde (APS), envolvendo indicadores de acesso, resolutividade e procedimentos preventivos realizados. Foram incluídos, também, indicadores de organização da rede de atenção à saúde bucal e sociodemográficos. No nível estadual, por sua vez, foram mensurados Índice de Desenvolvimento Humano (IDH) e Gini^2,6,8,13^. As covariáveis do nível municipal e estadual foram quantitativas, exceto porte populacional (categórica ordinal) e existência de CEO (dicotômica). As descrições e fontes de dados das covariáveis estão detalhadas no Material Suplementar 1.

### Análise estatística

Inicialmente, foram realizadas análises descritivas do desfecho e das covariáveis em níveis individual, municipal e estadual. Modelos de regressão de Poisson não ajustados, com variância robusta, foram desenvolvidos para testar a associação entre as variáveis independentes no nível individual e a perda dentária, como variável binária. As razões de prevalência (RP) não ajustadas (intervalos de confiança de 95% – IC95%) e os valores de p foram estimados. Para a checagem da necessidade de desenvolvimento de modelos de regressão multinível, o modelo nulo foi desenvolvido. A análise de regressão de Poisson multinível foi utilizada para estimar as RP e o IC95% entre as variáveis individuais, municipais e estaduais ao desfecho binário. Modelos de regressão de Poisson com variância robusta são uma opção para estimar RP em estudos transversais com a variável dependente binária^17^. O primeiro modelo incluiu variáveis individuais com p<0,20 nos modelos de regressão não ajustados; o segundo, variáveis municipais e as covariáveis individuais com p<0,20. Já o modelo final incluiu as covariáveis estaduais, mantendo aquelas com p<0,20 nos modelos anteriores.

A variância explicada nos níveis municipal e estadual foi estimada pelo coeficiente de partição da variância, comparando o modelo final ao modelo nulo. As análises primárias foram desenvolvidas com aqueles indivíduos que apresentaram todos os dados presentes. Desenvolveram-se, ainda, análises de sensibilidade incluindo as categorias não sabe/não respondeu para cada covariável do nível individual. As análises estatísticas, considerando pesos amostrais, foram desenvolvidas no SPSS 23.0 e *STATA* 12.0 (College Station, TX, EUA).

### Aspectos éticos

O Projeto SB Brasil 2023 seguiu a Resolução n^o^ 466/2012, do Conselho Nacional de Saúde (CNS), e recebeu aprovação da Comissão Nacional de Ética em Pesquisa (Conep), sob CAAE 34497120.6.3001.0008 e parecer n^o^ 4.823.054, com assinatura do Termo de Consentimento Livre e Esclarecido (TCLE) pelos participantes.

#### Declaração de Disponibilidade de Dados:

Dados disponíveis mediante solicitação.

## RESULTADOS

A descrição das condições demográficas e da morbidade bucal dos 8.053 adolescentes participantes do inquérito nacional SB Brasil 2023 está descrita na Tabela 1. As variáveis contextuais de nível municipal e estadual estão descritas nos Materiais Suplementares 2 e 3, respectivamente. A prevalência de perda dentária foi de 13,77% (IC95% 11,93–15,85). Na análise não ajustada, a perda foi mais frequente entre não brancos, beneficiários de programas de assistência social, adolescentes com dor de dente nos últimos seis meses e usuários do serviço público odontológico (p<0,05) (Tabela 2).

**Tabela 1:** Frequência das variáveis independentes individuais pesquisadas entre adolescentes brasileiros de 15 a 19 anos (n=8.053), 2023.

Dimensão		Variável	n	% (IC95%)
>Sociodemográficas	Sexo			
		Feminino	4.178	49,81 (46,89–52,72)
		Masculino	3.875	50,18 (47,27–53,10)
	Raça/Cor			
		Branco	2.604	37,73 (33,11–42,58)
		Não branco	5.359	61,51 (56,69–66,12)
		Não sabe/não respondeu*	90	0,75 (0,42–1,31)
	Formas de acesso à água			
		Canalizada	7.438	93,97 (92,06–95,45)
		Não canalizada	226	2,67 (1,73–4,09)
		Não sabe/não respondeu*	389	3,34 (2,29–4,84)
	Acesso à internet			
		Não	349	3,27 (2,49–4,28)
		Sim	7.385	93,43 (91,58–94,90)
		Não sabe/não respondeu*	319	3,29 (2,23–4,81)
	Beneficiário de programas de assistência social			
		Não	4.030	50,44 (46,25–54,62)
		Sim	3.293	41,51 (37,65–45,48)
		Não sabe/não respondeu*	609	8,03 (5,33–11,94)
	Cobertura por plano de saúde odontológico			
		Não	6.997	86,25 (82,08–89,57)
		Sim	910	12,03 (8,83–16,19)
		Não sabe/não respondeu*	146	1,70 (0,61–4,64)
			**Média (IC95%)**	
	Idade (anos)		16,85 (16,77–16,93)	
	Escolaridade (anos)		11,03 (10,62–11,45)	
>Morbidade bucal	Dor de dente nos últimos 6 meses			
		Não	6.289	77,70 (74,79–80,36)
		Sim	1.708	20,93 (18,59–23,49)
		Não sabe/não respondeu*	56	1,35 (0,57–3,16)
Utilização de serviços	Tipo de serviço utilizado			
		Outros (privado, planos, outros)	3.358	45,10 (41,18–49,08)
		Público	3.784	43,91 (40,59–47,28)
		Nunca usou	480	4,59 (3,63–5,79)
		Não sabe/não respondeu*	431	6,38 (4,01–10,01)

95%IC: Intervalo de confiança de 95%.

* A categoria "Não sabe/não respondeu" foi considerada dado perdido nos modelos subsequentes.

**Tabela 2:** Fatores individuais associados à perda dentária entre adolescentes brasileiros de 15 a 19 anos, 2023.

Dimensão	Variável		Perda dentária (%, IC95%)	RP Bruta	p-valor
Sociodemográficas	Sexo				
		Feminino	13,88 (11,48–16,70)	1	0,642
		Masculino	13,67 (10,84–17,11)	0,97 (0,87–1,09)	
	Raça/Cor				
		Branco	9,42 (7,27–12,12)	1	<0,001
		Não branco	16,57 (14,16–19,29)	1,35 (1,17–1,56)	
	Formas de acesso à água				
		Canalizada	13,54 (11,66–15,66)	1	0,013
		Não canalizada	16,86 (9,47–28,21)	1,47 (1,08–1,98)	
	Acesso à internet				
		Não	17,50 (11,81–25,14)	1	0,005
		Sim	13,54 (11,64–15,70)	0,70 (0,55–0,90)	
	Programas de assistência social				
		Não	10,33 (8,22–12,91)	1	<0,001
		Sim	17,79 (14,99–20,97)	1,43 (1,26–1,63)	
	Cobertura por plano de saúde				
		Não	14,60 (12,59–16,87)	1	0,009
		Sim	8,77 (5,87–12,92)	0,75 (0,61–0,93)	
	Idade			1,18 (1,13–1,23)	<0,001
	Escolaridade			1,00 (0,99–1,01)	0,822
Morbidade bucal	Dor de dente nos últimos 6 meses				
		Não	10,67 (9,11–12,47)	1	<0,001
		Sim	24,75 (19,01–31,54)	1,89 (1,67–2,15)	
Utilização de serviços	Outros (privado, planos)		13,92 (7,54–24,29)	1	
		Público	17,50 (14,47–21,02)	1,37 (1,21–1,56)	<0,001
		Nunca usou	10,07 (7,96–12,65)	0,65 (0,47–0,91	0,013

95%IC: Intervalo de confiança de 95%; RP: Razão de prevalência.

O modelo multinível com variáveis individuais, municipais e estaduais foi desenvolvido com 6.854 adolescentes (taxa de perda por incompletude dos dados=14,9%). Os achados revelaram que indivíduos beneficiários de programas sociais apresentavam uma prevalência 25% maior de perda dentária em relação aos não beneficiários (RP 1,25; IC95% 1,10–1,43). O aumento de um ano na idade aumenta em 19% a prevalência de perda dentária (RP 1,19; IC95% 1,13–1,24). Presença de dor de dente aumenta em 66% a prevalência de perda dentária neste grupo (RP 1,66; IC95% 1,45–1,90). Usuários do setor público têm uma prevalência 23% maior de perda dentária do que aqueles que usam o setor privado (RP 1,23; IC95% 1,07–1,41). Adolescentes que nunca utilizaram os serviços de saúde bucal têm prevalência 43% menor de perda dentária (RP 0,57; IC95% 0,39–0,83).

Em nível municipal, o aumento de 1% na cobertura de saúde bucal na atenção primária diminui a prevalência de perda dentária em aproximadamente 0,5% (RP 0,99; IC95% 0,99–0,99). O aumento do Produto Interno Bruto (PIB) municipal diminui a prevalência de perda dentária (RP 0,99; IC95% 0,99–1,00). A prevalência de perda dentária aumenta com a diminuição do porte populacional, sugerindo a existência de um gradiente de dose-resposta. Estados com maior IDH diminuem a prevalência de perda dentária entre adolescentes brasileiros (RP 0,04; IC95% 0,00–0,98).

A avaliação das mudanças das variâncias nos diferentes modelos em comparação ao modelo nulo nos permite afirmar que a inclusão gradativa das variáveis nos modelos 1, 2 e 3 melhora o percentual de explicação da perda dentária pelos fatores selecionados. Pode-se observar que a inclusão das variáveis dos Níveis II e III concomitantemente explica aproximadamente 100 e 72,04%, respectivamente, da variância (Tabela 3).

**Tabela 3:** Fatores individuais, municipais e estaduais associados à perda dentária entre adolescentes de 15 a 19 anos, Brasil, 2023.

Nível I – Indivíduos							
Variável		Modelo 1		Modelo 2		Modelo 3	
		RP (IC95%)	p-valor	RP (IC95%)	p-valor	RP (IC95%)	p-valor
Raça/Cor							
	Branco	1	0,025	1	0,019	1	0,055
	Não branco	1,21 (1,02–1,42)		1,21 (1,03–1,43)		1,17 (1,00–1,37)	
Formas de acesso à água							
	Canalizada	1	0,189	1	0,261		
	Não canalizada	1,26 (0,89–1,77)		1,21 (0,87–1,70)			
Acesso à internet							
	Não	1	0,082	1	0,132	1	0,059
	Sim	0,79 (0,60–1,03)		0,81 (0,62–1,06)		0,77(0,59–1,01)	
Beneficiário de programas de assistência social							
	Não	1	0,001	1	0,002	1	0,001
	Sim	1,27 (1,11–1,45)		1,24 (1,09–1,42)		1,25 (1,10–1,43)	
Cobertura por plano de saúde odontológico							
	Não	1	0,161	1	0,228		-
	Sim	0,84 (0,66–1,07)		0,86 (0,68–1,10)			
Idade		1,19 (1,13–1,24)	<0,001	1,18 (1,13–1,24)	<0,001	1,19 (1,14–1,24)	<0,001
Dor de dente nos últimos 6 meses							
	Não	1	<0,001	1	<0,001	1	<0,001
	Sim	1,67 (1,45–1,91)		1,66 (1,45–1,90)		1,66 (1,45–1,90)	
Tipo de serviço utilizado							
	Demais tipos de serviço (privado, planos, outros)	1		1		1	
	Público	1,22 (1,06–1,41)	0,006	1,22 (1,06–1,41)	0,007	1,23 (1,07–1,41)	0,003
	Nunca usou	0,59 (0,40–0,87)	0,007	0,58 (0,39–0,84)	0,005	0,57 (0,39–0,83)	0,004
**Nível II – Municípios**							
Primeira consulta		-	-	0,98 (0,96–1,00)	0,072	0,98 (0,96–1,00)	0,062
TC/PC		-	-	0,99 (0,99–1,00)	0,310		
Procedimentos preventivos		-	-	0,99 (0,99–1,00)	0,133	0,99 (0,99–1,00)	0,141
Cobertura de SB na APS		-	-	0,99 (0,99–1,00)	0,064	0,99 (0,99–0,99)	0,038
Existência de CEO							
	Não	-	-	1			
	Sim	-	-	0,95 (0,87–1,04)	0,263		
	PIB per capita	-	-	0,99 (0,99–0,99)	0,002	0,99 (0,99–1,00)	0,036
Porte populacional (mil)							
	>500	-	-	1		1	
	>100 e ≤500	-	-	1,22 (0,95–1,57)	0,114	1,32 (1,08–1,63)	0,007
	>10 e ≤ 100			2,06 (1,33–3,20)	0,001	2,33 (1,70–3,20)	<0,001
	Até 10	-	-	2,32 (1,25–4,31)	0,008	2,61 (1,62–4,21)	<0,001
**Nível III – Estados**							
IDH		-	-	-	-	0,04(0,00–0,98)	0,048
Gini		-	-	-	-	5,43(0,44–68,74)	0,188
**Efeitos randômicos**		**Modelo Nulo Nível II**	**Modelo Nulo Nível III**	**Modelo Nulo Níveis II e III**	**Modelo 1**	**Modelo 2**	**Modelo 3**
Variância (IC95%)		0,217 (0,130–0,362)	0,127 (0,065–0,250)	II: 0,099 (0,044–0,223) III 0,142 (0,066–0,309)	II: 0,058 (0,017–0,204) III: 0,098 (0,041–0,236)	II: 0,000 (0,000–0,000) III: 0,063 (0,025–0,158)	II: 0,000 (0,000–0,000) III: 0,039 (0,014–0,110)
LR test (χ^2^; p-valor)		74,53 (<0,001)	87,51 (<0,001)	100,07 (<0,001)	46,71 (<0,001)	21,86 (<0,001)	12,35 (0,002)
Mudanças na variação em relação ao modelo nulo com os níveis II e III					II: 41,47% III: 31,19%	II: 99,78% III: 57,62%	II: 100% III: 72,04%

RP: razão de prevalência; IC: Intervalo de confiança; TC/PC: Proporção de tratamentos concluídos em relação às primeiras consultas; SB: saúde bucal; APS: Atenção Primária à Saúde; CEO: Centros de Especialidades Odontológicas; IDH: Índice de Desenvolvimento Humano; LR: *likelihood ratio*.

A análise de sensibilidade realizada com a inclusão da categoria não sabe/não respondeu identificou a permanência de associações com as variáveis idade, ser beneficiário de programa social, dor de dente, tipo de serviço utilizado e porte do município — com direção dessas associações mantidas e magnitude das associações próximas ao modelo apresentado nos resultados primários. Houve, ainda, inclusão de novas associações (acesso à internet e indicador de primeira consulta odontológica programática) e a perda de associação (cobertura de SB na APS, PIB e IDH — que tinha uma associação marginal no modelo original com p=0,048). Além disso, houve no modelo 3 (final) modificação no percentual de variância explicada para aproximadamente 95,75 e 76,24%, respectivamente (Material Suplementar 4).

## DISCUSSÃO

Este estudo multinível de base populacional identificou determinantes individuais e contextuais da perda dentária em adolescentes brasileiros de 15 a 19 anos. A prevalência da perda dentária permanece frequente nessa faixa etária, indicando um agravo precoce e potencialmente evitável, determinada, principalmente, por fatores sociodemográficos, socioeconômicos, de morbidade bucal, utilização dos serviços, individuais e contextuais.

No nível individual, a dor de dente destacou-se como fator relevante, aumentando em 66% a prevalência de perda dentária e atuando como marcador da gravidade da cárie e motivador da procura por atendimentos odontológicos^18^. No contexto brasileiro, a percepção da dor e da perda dentária influencia o comportamento em saúde bucal^19^, estando associada a piores desfechos e a barreiras de acesso, especialmente em modelos de cuidado centrados em urgências, em detrimento de ações preventivas^11,19^.

O aumento de um ano na idade elevou em 19% a prevalência de perda dentária, corroborando Motta et al.^4^ e possivelmente refletindo maior gravidade da cárie em faixas etárias mais avançadas da adolescência, além de fatores culturais e de acesso aos serviços. Embora Drummond et al.^7^ apontem redução progressiva da perda dentária entre adolescentes nos inquéritos SB Brasil 2003, 2010 e 2023, provavelmente por conta de políticas públicas como o Programa Brasil Sorridente e a Estratégia Saúde da Família (ESF), os presentes achados sugerem que esse desfecho ainda persiste, com acúmulo precoce de agravos bucais e persistência de desigualdades regionais e socioeconômicas.

A literatura sobre a saúde bucal entre beneficiários de programas de transferência de renda, como o Bolsa Família^20^, ainda é escassa. Adolescentes beneficiários apresentaram 25% maior prevalência de perda dentária, reforçando a influência das desigualdades socioeconômicas. Colvara et al.^21^ observaram tendência de maior número de exodontias em municípios com maior cobertura do programa. Esses achados evidenciam a necessidade de estudos que avaliem essa associação entre diferentes faixas etárias e a persistência de barreiras estruturais e desigualdades no acesso à saúde bucal.

Apesar da ampliação das políticas públicas, como a Política Nacional de Saúde Bucal (PNSB), conhecida como Brasil Sorridente; e da ESF, com a incorporação das Equipes de Saúde Bucal (eSB), dos programas sociais e da expansão dos serviços odontológicos, o acesso e uso dos serviços odontológicos continuam desiguais^22^. Essa desigualdade é evidenciada pela associação entre utilização dos serviços e perda dentária entre adolescentes, mediada, principalmente, por condições socioeconômicas individuais^13,23^.

No presente estudo, usuários do setor público apresentaram 23% maior prevalência de perda dentária, enquanto aqueles que nunca utilizaram serviços odontológicos apresentaram 43% menor prevalência. Esses resultados devem ser interpretados com cuidado, pois a menor prevalência entre adolescentes que nunca utilizaram serviços odontológicos pode refletir um padrão de utilização orientado pela necessidade, compatível com o viés do indivíduo saudável, no qual indivíduos com melhores condições de saúde bucal tendem a demandar menos os serviços de saúde^8,24^.

Tais achados sugerem que o uso de serviço público ocorre, em geral, diante de necessidades acumuladas, com busca predominante por dor, urgência e/ou demanda curativa, e não por ações preventivas, refletindo um modelo historicamente centrado em atendimentos de urgência e emergência^21^. Estudos prévios sustentam esse achado, como o de Motta et al.^4^, no qual adolescentes atendidos pelo SUS relataram maior frequência de consultas motivadas por dor; enquanto no estudo de Oliveira et al.^10^ destacou-se que o uso do SUS está associado a condições socioeconômicas mais desfavoráveis e menor acesso a cuidados preventivos.

Adolescentes residentes em municípios com maior cobertura de saúde bucal na APS apresentaram menor prevalência de perda dentária, reforçando a eficácia das ações preventivas e de promoção da saúde nesse nível de atenção. Esse achado reforça a atuação estratégica da APS como coordenadora do cuidado e porta de entrada do SUS. Estruturada majoritariamente por meio da ESF e pelas eSB, a APS exerce atuação essencial na prevenção da cárie e na redução de suas consequências, como a perda dentária^25-27^. Dessa forma, a maior cobertura da APS reflete maior capacidade dos municípios em organizar respostas e ações preventivas, reduzir desigualdades e promover integralidade do cuidado.

É importante ressaltar que os resultados encontrados no nível individual (usuários do setor público) não se contrapõem aos achados dos indicadores contextuais (como a cobertura da APS), uma vez que cada um reflete mecanismos diferentes. O uso do serviço público tende a refletir as necessidades instaladas, enquanto a cobertura da APS e outros fatores municipais expressam a capacidade organizacional do território de ofertar ações preventivas e coordenar o cuidado. A categoria "nunca usou o serviço odontológico" deve ser interpretada com cautela, pois pode refletir tanto ausência de necessidade percebida quanto barreiras de acesso aos serviços, influenciando as associações observadas.

O aumento do PIB municipal diminuiu a prevalência de perda dentária, sugerindo que o desenvolvimento econômico local pode proporcionar melhores condições de vida e acesso a recursos de saúde. Entretanto, esse resultado deve ser interpretado com cautela. Como indicador macroeconômico agregado, o PIB não capta a distribuição de renda no território, podendo coexistir como concentração de riqueza e desigualdades no acesso aos serviços^28,29^. Dessa forma, o achado pode refletir características estruturais do território, mas não significa que há uma melhor distribuição ou qualidade do cuidado. Para uma interpretação mais abrangente, é necessário considerar outros indicadores de desigualdade.

No que se refere ao porte populacional dos municípios, aqueles de maior porte tendem a apresentar maior disponibilidade de serviços de saúde e de profissionais, fatores que facilitam o acesso aos cuidados em saúde bucal e contribuem para melhores desfechos^12^. Em contrapartida, municípios de pequeno porte frequentemente lidam com desafios relacionados à disponibilidade de recursos e à contratação de profissionais, o que tende a comprometer a oferta e qualidade da assistência odontológica^30^.

O IDH apresenta forte correlação com indicadores de saúde, demonstrando que o processo saúde-doença é também produto das condições de qualidade de vida^31,32^. Os resultados deste estudo indicaram que estados com maior IDH associaram-se a menor prevalência de perda dentária entre adolescentes brasileiros, sugerindo que melhores condições socioeconômicas e educacionais refletem em desfechos mais favoráveis de saúde bucal^9^. Estudos prévios realizados com adultos brasileiros identificaram menores médias de dentes perdidos e menores desigualdades socioeconômicas em municípios com maior IDH^9,33^. Contudo, por se tratar de um indicador agregado, o IDH estadual pode mascarar desigualdades intrarregionais e locais, indicando a necessidade de análises em nível municipal para melhorar a compreensão dos efeitos contextuais e territoriais associados à perda dentária^34^.

Entre as variáveis analisadas neste estudo, não foram observadas associações estatisticamente significativas (p<0,05) entre a perda dentária e as seguintes características individuais: forma de acesso à água, acesso à internet e cobertura por plano de saúde odontológico. Em nível municipal, a cobertura de primeira consulta odontológica, proporção de tratamentos concluídos, realização de procedimentos preventivos e existência de Centros de Especialidades Odontológicas (CEO) não apresentaram associação com o desfecho. Por fim, no nível estadual, o índice de Gini, que expressa a desigualdade de renda na população, também não mostrou associação significativa com a perda dentária entre adolescentes. Esse achado pode ser uma consequência positiva das políticas públicas de ampliação da atenção primária e da oferta mais equitativa de serviços odontológicos no país, que contribuem para reduzir as desigualdades regionais em saúde bucal. Estudos como os de Ferreira et al.^9^ e Roberto et al.^33^ apontam que o efeito cumulativo dos determinantes sociais tende a se intensificar em faixas etárias mais avançadas, o que explicaria a menor influência desses fatores na população de adolescentes entre 15 e 19 anos.

A perda dentária está fortemente associada a pior qualidade de vida, afetando atividades cotidianas e o bem-estar social, emocional e psicológico^3,35^. Na adolescência, o impacto é ampliado por se tratar de uma fase de maior vulnerabilidade, marcada por transformações biopsicossociais e comportamentos de risco, como tabagismo, etilismo e práticas inadequadas de higiene bucal, que podem se perpetuar na vida futura^14^.

Importante ressaltar que se trata de um estudo transversal, impossibilitando o estabelecimento de relações de causalidade entre as variáveis analisadas. Além disso, o uso de bancos de dados secundários pode implicar limitações relacionadas à presença de dados faltantes e ao eventual preenchimento incorreto de informações, mesmo os provenientes dos bancos oficiais do Sistema Único de Saúde. Destaca-se que o estudo utiliza dados provenientes do mais recente inquérito nacional de saúde bucal (SB Brasil 2023), o que possibilita a análise dos dados mais atuais acerca da saúde bucal da população no Brasil.

A análise de sensibilidade demonstrou que os achados devem ser interpretados com cautela, tendo em vista a modificação das variáveis associadas ao desfecho com a inclusão das categorias não sabe/não respondeu nas variáveis pertinentes, porém, as variáveis com associações mais consistentes se mantiveram no modelo original e nesta análise de sensibilidade — o que pode ser considerado um ponto positivo para os resultados primários. Até o momento, não foram identificados estudos que avaliem a perda dentária em adolescentes brasileiros de 15 a 19 anos considerando fatores individuais e contextuais com base nos dados nacionais mais recentes. Os resultados são relevantes, pois fornecem uma visão nacional abrangente e atualizada, contribuindo para a avaliação dos serviços e o aprimoramento de políticas públicas voltadas à ampliação do acesso e melhoria da assistência odontológica no país.

Por fim, a perda dentária entre adolescentes de 15 a 19 anos permanece como um importante agravo em saúde pública, fortemente influenciado por condições sociodemográficas, de morbidade bucal, de acesso aos serviços odontológicos, e contextuais. Os resultados apontam a urgência de gestores e governos fortalecerem ações de promoção e prevenção, ampliarem o acesso aos cuidados odontológicos, focarem em territórios com maiores desigualdades e priorizarem políticas públicas mais equitativas e integradas. Estudos futuros devem comparar esses achados com outras faixas etárias e períodos, contribuindo para o acompanhamento das tendências temporais de saúde bucal no país.

## Material suplementar

Material Suplementar
